# Cortical control of posture in fine motor skills: evidence from inter-utterance rest position

**DOI:** 10.3389/fnhum.2023.1139569

**Published:** 2023-08-17

**Authors:** Eric Easthope, Arian Shamei, Yadong Liu, Bryan Gick, Sidney Fels

**Affiliations:** ^1^Human Communication Technologies Lab, Department of Electrical and Computer Engineering, University of British Columbia, Vancouver, BC, Canada; ^2^Integrated Speech Research Lab, Department of Linguistics, University of British Columbia, Vancouver, BC, Canada; ^3^Haskins Laboratories, New Haven, CT, United States

**Keywords:** sensorimotor beta, sensorimotor gamma, speech motor control, fine motor skill, posture, postural control, electrocorticography, inter-utterance rest position

## Abstract

The vocal tract continuously employs tonic muscle activity in the maintenance of postural configurations. Gamma-band activity in the sensorimotor cortex underlies transient movements during speech production, yet little is known about the neural control of postural states in the vocal tract. Simultaneously, there is evidence that sensorimotor beta-band activations contribute to a system of inhibition and state maintenance that is integral to postural control in the body. Here we use electrocorticography to assess the contribution of sensorimotor beta-band activity during speech articulation and postural maintenance, and demonstrate that beta-band activity corresponds to the inhibition of discrete speech movements and the maintenance of tonic postural states in the vocal tract. Our findings identify consistencies between the neural control of posture in speech and what is previously reported in gross motor contexts, providing support for a unified theory of postural control across gross and fine motor skills.

## 1. Introduction

A growing body of electrocorticography (ECoG) research on speech and non-speech movements has identified gamma (γ) and high-frequency γ (Γ) oscillations with the control of transient movement in the sensorimotor cortex (SMC) (Szurhaj and Derambure, 2006; Bouchard et al., 2013; Chartier et al., 2018; Ulloa, 2022). Initially only lower-frequency γ (35–60 Hz) activity was reported, but later intracranial studies identified higher-frequency γ (60–200 Hz) contributions to movement (Abbs, 1973; Bouchard et al., 2013; Ulloa, 2022). Spoken phonemes, syllables, and words can be discriminated from activations in the SMC, which is somatotopically organized (Bouchard et al., 2013; Ramsey et al., 2018). This work is based on the premise that spikes in γ and Γ unambiguously distinguish speech movements of articulators from their resting state (Chartier et al., 2018). Paradoxically, it is known that tonic muscle activity is continuously employed in the vocal tract for maintenance of inter-utterance and clinical rest postures, such that musculature in the vocal tract is never truly at rest while awake, yet the neural oscillations that modulate this activity remain unknown. While there has been substantial research on γ and Γ contributions to the control of transient speech movements, there has been comparatively little research on the contributions of other waves to speech motor control and the control of tonic muscle activation in the vocal tract (Salari et al., 2018). Here we compare sensorimotor beta (β) oscillations with γ and Γ oscillations during speech and inter-utterance rest to understand β contributions to speech motor control and postural control of vocal tract articulators.

Among neural oscillations, β waves are the least understood with ongoing debate over their function (Engel and Fries, 2010; Kilavik et al., 2013; Chandrasekaran et al., 2019; Schmidt et al., 2019). There is substantial evidence that sensorimotor beta-band activity (BBA; 12–35 Hz) contributes to motor function throughout the body, namely in observations that BBA increases at the termination of movement and maintenance of a steady state (“beta rebound”), and decreases at onset of movement (“beta desynchronization”) (Kilavik et al., 2013). However, there is still ongoing disagreement even within the domain of motor control as to whether BBA in the SMC represents a system of inhibition and motor set maintenance or a system that predicts upcoming changes to state. This has philosophically manifested as a debate over whether BBA modulates posture or movement planning (for a review, see Engel and Fries, 2010; Schmidt et al., 2019), resulting from ambiguity in whether increased BBA corresponds to the termination of the previous movement and sustained maintenance of the current motor set, and/or planning of the subsequent movement. For example, in gross motor contexts there is strong evidence that BBA is linked to control of posture and tonic muscle activations throughout the body; β oscillations are coherent and synchronized to tonic muscle contractions (Baker et al., 1997) and correspond to the degree of postural complexity defined as the muscular activity necessary to counteract gravity (Tia and Pifferi, 2021). Yet, evidence for the role of BBA in movement planning is strong as well; BBA appears to correspond to the complexity of upcoming motor tasks in non-speech settings (Tzagarakis et al., 2010; Heinrichs-Graham and Wilson, 2015), and BBA is modulated by response uncertainty in stimuli selection tasks (Palmer et al., 2016). Thus, it remains unclear whether BBA contributes to a system of motor inhibition and state maintenance, motor planning, or both.

To our knowledge two previous works have examined the role of BBA in the SMC in speech motor control. Livezey et al. (2019) used deep learning models to evaluate speech motor control via SMC ECoG during articulation of individual syllables, but their analysis focused on classification accuracy of speech signals and thus did not provide a detailed account of beta-band event-related potentials (ERPs) during speech production. However, they report a significant positive Γ–β correlation near consonant-vowel transitions. This is unexpected in motor control contexts where negative Γ–β correlations (anti-correlations) are typically observed; Livezey et al. note the positive correlation is band-limited to specific beta-band frequencies and hence it may have been previously overlooked.

More recently De Nil et al. (2021) compared BBA via magnetoencephalography (MEG) between simple (e.g., /pa pa pa/, single-finger button task) and complex (e.g., /pa ka ta pa/, multi-finger button task) verbal and manual motor task sequences, and report the presence of beta desynchronization during movement planning and beta rebound after movement termination in both speech and non-speech motor tasks. They observe transient partial re-synchronization during the “movement planning stage” for simple but not complex task sequences, which suggests that BBA is modulated by the complexity of upcoming movement sequences; complex sequences also exhibit greater beta rebound than simple sequences at movement termination, suggesting that β oscillations are sensitive to the complexity of the preceding task.

While this work helps unify our understanding of BBA across speech and non-speech contexts, it is still not known whether BBA modulates movement inhibition and/or motor set preservation in speech motor control. De Nil et al. (2021) report the presence of beta desynchronization beginning more than three seconds before movement and terminating after movement ends, but they do not analyze how BBA varies in response to individual movements within a sequence. In non-speech motor contexts, such as with arm movements, it is known that movement onset itself should result in a measurable reduction in BBA distinct from any changes in earlier planning stages (Stancák and Pfurtscheller, 1995; Erbil and Ungan, 2007). This observation is crucial to theories of BBA as a system of movement inhibition and state maintenance (Engel and Fries, 2010; Kilavik et al., 2013). Yet De Nil et al. (2021) report no changes to BBA after onset of initial movement in both speech and hand movement sequences, which is unexpected based on previous accounts of BBA during movement. Furthermore, it is still not known whether BBA in speech motor control, as a fine motor skill, corresponds to tonic muscle activity and postural complexity as is observed in gross motor contexts (Tia and Pifferi, 2021). Notably, in gross motor contexts it has been suggested that β oscillations may be composed of several distinct co-occurring rhythms each corresponding to distinct motor functions (Kilavik et al., 2013). For example, lower-frequency β has been described as “anti-kinetic” whereas higher-frequency β may correspond more to movement planning, yet such a distinction remains unclear. To date, we know of no work investigating whether distinct sub-bands of β can be observed in fine motor skills. A unified theory of motor control would predict similar approaches to postural control across both fine and gross motor skills, and naturally this extends to control mechanisms for posture during execution of fine motor skills such as speech. Correspondingly any investigation of β in speech motor control should decompose β into relevant sub-bands identified with motor inhibition, maintenance, and/or planning.

Posture, as known since the time of the ancient Greeks, consists of tonic muscle activations which counteract the effects of gravity (Ivanenko and Gurfinkel, 2018). Until recently, posture was viewed as a component of gross motor skills, such as walking and balancing, and the control of posture was believed to be relegated to subcortical structures such as the brain stem, cerebellum and localized spinal neuronal circuits. We have since come to understand that there are substantial cortical contributions to the planning and execution of posture (Kilavik et al., 2013; Tia and Pifferi, 2021) and that tonic muscle activations which counteract gravity are essential to execution of fine motor skills in speech and non-speech contexts. For example, recent reports demonstrate impaired manual dexterity in astronauts after adaptation to microgravity, revealing that fine motor skills are sensitive to gravitational transitions (Holden et al., 2022; Weber et al., 2022). Similarly, microgravity adaptation impaired upward movement of the tongue during speech produced by astronauts immediately after their transition to Earth gravity (Shamei and Gick, 2019; Shamei et al., 2023). Contemporary models of speech motor control are naive to external factors such as gravity and focus primarily on transient movement. Presumably this is because gravitational effects are small and difficult to measure (for a review of contemporary models, see Parrell et al., 2019). However, the observation that gravitational transitions impair speech necessitates that any realistic speech motor control model has some mechanism to adaptively account for gravity. The similar effect of gravitational transitions on fine motor skills to those observed in gross motor contexts suggests a unified approach to the control of posture across fine and gross motor domains.

Speech is an ideal system for studying the use of posture and tonic motor activity in fine motor skills; tonic muscle activations are employed throughout speech, and vocal tract articulators return to a distinct inter-utterance speech posture (ISP, also known as inter-utterance rest position or inter-speech posture). The ISP is a language-specific postural configuration maintained between utterances by articulators such as the tongue, jaw, and velum, and is mechanically advantageous for the motor demands of a language (Gick et al., 2004; Ramanarayanan et al., 2014; Gick and Mayer, 2019). The movement trajectories and accuracy of the tongue as it enters ISP are similar to those during execution of speech movements, suggesting similar neural control mechanisms for movement to phonemic targets and ISP. However, maintenance of the ISP involves tonic muscle activity distinct from that employed during transient movement (Gick and Mayer, 2019). While transient movement is essential to move the tongue into ISP, tonic muscle activity is necessary to maintain this state. Previous ECoG research has identified γ and Γ oscillations with the control of transient speech movement, usually based on comparisons of speech movement to brief periods of inter-utterance rest, but it is still not known which oscillations correspond to mechanisms underlying tonic muscle activity during postural maintenance. Recent work by Salari et al. (2018) investigated the control of tonic muscle activations during sustained vowel phonations and found correspondences with sustained gamma-band activity (GBA) in Γ; this identification of sustained GBA with underlying tonic muscle activity during sustained articulation was also based on comparisons to inter-utterance rest. Crucially, if sustained γ and/or Γ oscillations corresponded to the control of tonic muscle activity during postural maintenance, we would expect abundant reports of sustained gamma synchronization during maintenance of a steady postural state. Yet the literature is consistent that GBA decreases during maintenance of a steady state where BBA simultaneously increases. Naturally, we expect that the control of postural state is modulated by neural activity present at or near the time when control occurs.

A system of transient movement into a tonic state—as described for ISP—is similar to how posture would be controlled in theories that attribute BBA to maintenance of status quo (Engel and Fries, 2010; Kilavik et al., 2013). In such models, once the desired posture is achieved via γ and/or Γ-modulated movements, β oscillations increase to inhibit further γ and/or Γ-modulated actions and to preserve the existing motor set. We hypothesize that sensorimotor BBA during speech reflects a system of motor inhibition and set maintenance compatible with status quo theories of β oscillations in motor and postural control; naturally, we also hypothesize that movement into ISP is modulated by γ and Γ while maintenance of ISP is modulated by β. In our analysis we compare neural control signals (β, γ, and Γ oscillations) corresponding with the execution of three motor tasks: (1) movement during execution of a syllable, (2) movement during return to ISP, and (3) maintenance of ISP. Based on these hypotheses we make several predictions:

1. **The onset of movement will result in measurable event-related desynchronization (ERD) of** **β**. BBA should decrease at movement onset. This contrasts with ERD being maintained from earlier movement planning stages as described by De Nil et al. (2021). While De Nil et al. report no change to BBA at movement onset for individual movements within a sequence, we expect to observe decreasing BBA at movement onset in a manner consistent with other fine motor skills.
  It is important to note that the articulation of a single syllable is usually the product of multiple movements. For example, in the articulation of the syllable “bee” there are (at least) three distinct sets of movements. First there is a bilabial consonant involving simultaneous movements of the lips, velum, tongue, and larynx, followed by a high vowel resulting from different motor demands of the same articulatory set, and finally the return of articulators to a distinct ISP configuration. Thus, a single syllable necessitates the execution and termination of several movements. De Nil et al. (2021) evaluated β modulation across sequences of four distinct syllables and noted no change in β across the entire sequence aside from initial desynchronization in the planning stage. However, a single syllable from such a sequence should produce several movements and correspondingly decreased BBA at the onset of each movement.
2. **Movement termination will result in transient event-related synchronization (ERS)**. As each syllable is the product of multiple movements, we expect measurable increases in BBA at the termination of each movement. For example, terminating a consonant movement should necessitate some degree of inhibition before executing another subsequent vowel movement. Likewise, terminating a vowel movement should also necessitate inhibition before subsequent return to ISP.
3. **Maintenance of ISP necessitates sustained ERS**. While we expect to see transient ERS several times throughout articulation of syllables where movement inhibition is necessary, we should also observe increased BBA sustained during times where a static tonic state is maintained. After vowel movement termination and subsequent return to ISP we expect to see the strongest ERS maintained until ISP is terminated at the onset of subsequent movement.
4. **Movement to ISP corresponds to** γ **and** **Γ** **oscillations**. We expect consistencies between movement trajectories of phonemes and return to ISP to reflect consistencies of the underlying neural control mechanisms, which are identified as γ and Γ for phonemic targets (Chartier et al., 2018; Ramsey et al., 2018; Salari et al., 2018; Livezey et al., 2019). Support for unified control underlying the execution of phonemes and return to ISP is reported by Chartier et al. (2018) and Salari et al. (2018, 2019), who observed distinct Γ spikes corresponding to vowel articulation and subsequent return to inter-utterance rest; Γ oscillations that would correspond to return to ISP occurred near vowel movement termination. This effect was robust across speech tasks involving sustained vowels as well as vowels repeated quickly in sequence, so we expect to see Γ ERS at vowel termination to return to ISP.
5. β **and** γ/Γ **will be anti-correlated during movement and ISP**. Based on observations of both gross and fine motor skills throughout the body, we expect that β and γ/Γ will be negatively correlated during movement execution and maintenance of tonic postural state. While there has been little work that explicitly evaluates correspondences of sensorimotor β and γ/Γ during speech motor control, we re-iterate that Livezey et al. (2019) observed a positive Γ–β correlation near consonant-vowel transitions. They note that this conflicts with previously observed β–γ anti-correlations in other motor control domains, but we think this correlation at consonant-vowel transition reflects BBA during inhibition of consonant movement in close proximity to GBA during execution of the subsequent vowel. Speech movements—especially consonant movements—are notably short (<100 ms). Thus, while we expect a general β–γ anti-correlation during inter-utterance rest and movement, we expect points of overlap between inhibitory BBA and GBA underlying movement termination and execution at transitional periods, especially during execution of shorter speech movements.

In sum, while there has been little work to evaluate the role of tonic muscle activity and β in speech motor control, previous descriptions of ISP involving transient movement into a tonically-maintained state are consistent with interpretations of BBA involving maintenance of status quo and more generally the control of posture. We expect to see a strong correspondence between the neural control of movement and postural maintenance in speech and what is observed in non-speech tasks. We also expect to see transient movement modulated by higher-frequency oscillations in γ and Γ, and inhibition and maintenance of tonic states by β.

## 2. Materials and methods

### 2.1. Dataset

Bouchard and Chang (2019) provide ECoG recordings of cortical surface electrical potentials (CSEPs) for four human subjects (sex unavailable) performing a syllable speech task, each with a high-density 256-channel (4 mm pitch) subdural ECoG array implanted over the left hemisphere perisylvian cortex. We chose ECoG for its high spatiotemporal resolution (Siero et al., 2014) and small but specific placement over sensorimotor areas of the brain. ECoG recordings captured amplified and digitally-processed continuous multi-channel CSEP data with annotated start, transition, and stop times for a trialed syllable speech production task. All subjects were native English speakers, had self-reported normal hearing, passed Boston Naming and verbal fluency tests, and showed no dysarthria. Subjects read aloud different consonant-vowel (CV) syllable combinations (one of 19 consonants, then one of 3 vowels: /a/, /i/, or /u/) and speech audio was recorded and synchronized to ECoG during CV production. The dataset contains 5.5 hours of 256-channel ECoG signals sampled at 3052 Hz over 31 sessions (3-14 per subject, 3-17 minutes per session) with session ECoG and annotations provided in NWB files (Teeters et al., 2015; Rübel et al., 2022). Session audio was not distributed for HIPAA compliance.

### 2.2. Data pre-processing

We used cortical locations labelled by Bouchard and Chang (2019) to identify SMC channels for ECoG electrodes implanted over the pre-central and post-central gyrus. We took these channels by index and excluded indices of invalid (“bad”) channels marked as having noise and/or seizure-related (*ictal*) artifacts during session recordings (for specific SMC indices included by subject, see Supplementary Table S1; for a visualization of SMC indices by subject, see Supplementary Figure S1). Bad indices were excluded from SMC indices by session (for specific bad indices excluded by session, see Supplementary Table S2), and we common average re-referenced remaining SMC channels by subtracting the average SMC channel signal from each channel.

Using re-referenced SMC channels and annotations from Bouchard and Chang (2019) for speech start, CV transition, and speech end times, as well as derived inter-speech times from adjacent speech end and start times, we made epochs of speech trial ECoG data from sequences of alternating speech (consonant, CV transition, and vowel) and ISP time intervals (ISP1-CV-ISP2; 11,024 total, 70–612 per session). We excluded the first and last trial per session where ISP1 and ISP2 are respectively undefined, and excluded trials with time intervals overlapping invalid times marked by Bouchard and Chang (2019) where unwanted ECoG artifacts would prevent us from making assured inferences about regular speech motor activity.

We kept trials with syllables ending in /i/ (pronounced “ee”), set a minimum ISP length (500 ms) to coarsely match the average CV length across subjects (~495 ms; 107 ms consonant; 388 ms vowel) and enable comparison with speech movement intervals, and excluded trials with ISP1 or ISP2 intervals shorter than 500 ms. This left us with signals for 2594 total ISP1-CV-ISP2 trials (18–161 per session) across 31 sessions, amounting to all trials with syllables ending in /i/ minus trials with undefined ISP intervals, unwanted ECoG artifact overlap, and insufficiently long ISP intervals. For longer ISP intervals we kept only 500 ms of each ISP interval nearest to each CV interval, plus an additional 500 ms of preceding and succeeding ISP signal to mitigate edge effects from convolution (from Hilbert transforms) during processing; most inter-speech intervals are ~500–1,000 ms long, so it is reasonable to interpret adjacent ISP2-ISP1 pairs as whole inter-utterance rests between subsequent syllables.

### 2.3. Data analysis

Trial signal epochs were notch filtered at 60 Hz and at harmonics 120 and 180 Hz to remove ECoG line noise artifacts. We applied a 3rd-order forward-backward digital Butterworth filter with cascaded second-order sections to bandpass useable ECoG channels into logarithmically-scaled sub-bands of β (β_ℓ_: 12–21 Hz and β_*h*_: 21–35 Hz), γ (γ_ℓ_: 35–50 Hz and γ_*h*_: 50–70 Hz) and Γ (Γ_ℓ_: 70–99 Hz and Γ_*h*_: 99–140 Hz). Frequency bandwidths and sub-band frequencies for β, γ, and Γ were informed by previous work and chosen to be compatible with ongoing investigations of β (Engel and Fries, 2010; Kilavik et al., 2013; Schmidt et al., 2019) and {γ, Γ} oscillations (Bouchard et al., 2013; Chartier et al., 2018; Ramsey et al., 2018; Salari et al., 2018; Livezey et al., 2019) in motor contexts.

After filtering we applied a Hilbert transform to each SMC channel to remove linear phase delay effects and to compute its analytic signal as a two-part complex signal *z*_*channel*_(*t*) = *A*_*channel*_(*t*) + *i* · θ_*channel*_(*t*) in terms of amplitude *A*_*channel*_ and phase θ_*channel*_. From this we computed the instantaneous bandpower per channel *P*_*channel*_(*t*) = $|z_{channel}\left(t\right){|}^{2}$ as the square of the time-varying analytic amplitude, computed total instantaneous bandpower *P*_*total*_(*t*) = $\underset{channel}{\sum }|z_{channel}\left(t\right){|}^{2}$ as the sum of all channel powers, and divided by the number of valid SMC channels *N*_*channels*_ to compute average instantaneous bandpower:


$$\begin{array}{l} P_{average}\left(t\right)=\frac{1}{N_{channels}}\underset{channel}{\sum }|A_{channel}\left(t\right)+i\cdot \theta_{channel}\left(t\right){|}^{2}. \end{array}$$

Similar to Bouchard et al. (2013) and Salari et al. (2018, 2019), we averaged instantaneous bandpower across {β_ℓ_, β_*h*_}, {γ_ℓ_, γ_*h*_}, and {Γ_ℓ_, Γ_*h*_} sub-bands to compute average β, γ, and Γ bandpowers within 12–35 Hz, 35–70 Hz, and 70–140 Hz respectively.

We truncated the additional 500 ms of preceding and succeeding ISP signal kept to mitigate edge effects from the average instantaneous bandpower *P*_*average*_(*t*) of each ISP1-CV-ISP2 trial, and interpolated *P*_*average*_(*t*) in consonant and vowel intervals with band-limited sinc interpolation to fix the length of variable CV intervals (consonant: 3–447 ms; vowel: 136–638 ms) across trials (Easthope, 2023); consonant and vowel signals were re-sampled over time to average trial consonant (~107 ms) and vowel lengths (~388 ms) respectively, enabling synchronized comparisons of neural activity across trials, sessions, and subjects without variability in speech start, CV transition, and speech end times.

We z-scored average instantaneous β, γ, and Γ bandpowers per session with respect to average session trial bandpower and standard deviation producing a derived dataset of z-scored fixed-length average instantaneous β, γ, and Γ bandpowers during ISP1-CV-ISP2 trials by all subjects, which we averaged across sessions to derive average trial {β_ℓ_, β_*h*_}, {γ_ℓ_, γ_*h*_}, and {Γ_ℓ_, Γ_*h*_} z-scores across all subjects.

To compare {β_ℓ_, β_*h*_}, {γ_ℓ_, γ_*h*_}, and {Γ_ℓ_, Γ_*h*_} sub-band powers during intervals of ISP preceding speech movement (ISP1), consonant movement, vowel movement, and ISP succeeding speech movement (ISP2) we computed Pearson product-moment correlation coefficients (r-values from normalized covariance in [−1, 1]) between z-scored sub-band pairs. For each *r*-value we computed a corresponding *p*-value for a two-tailed null hypothesis test that sub-band powers are uncorrelated and normally distributed; *p*-values approximate the probability of uncorrelated sub-bands having equal or greater *r*-values. Fifteen unique *r*-values were tested per interval so we compared *p*-values to a significance level α: = 0.05 with a Bonferroni correction *m* = 15 such that we compared their significance to α_*critical*_: = α/*m*≈0.003.

We also found local {β_ℓ_, β_*h*_}, {γ_ℓ_, γ_*h*_}, and {Γ_ℓ_, Γ_*h*_} sub-band maxima and minima during ISP1, consonant, vowel, and ISP2 intervals to identify extreme points over the time course of individual speech movements and inter-utterance rests, and wavelet transforms were computed per sub-band using Gaussian wavelets of varying width (standard deviation; σ = 1–42) to identify correlations and anti-correlations of Gaussian wavelets—resembling local maxima (“peaks”) and minima—to sub-band power oscillations over time. The standard deviations of time-domain Gaussian wavelets are inversely proportional to the standard deviations of their frequency-domain counterparts, so we chose a range of standard deviations (σ = 1–42) with frequency responses that span the entire sub-band frequency range (12–140 Hz) (for a comparison and demonstration of equivalencies between Fourier, Hilbert and wavelet-based spectral analysis, see Bruns, 2004).

To identify significant change points in sub-band powers over time we used Pruned Exact Linear Time multiple change-point detection with a Modified Bayesian Information Criterion penalty to find time intervals where sub-band statistics are relatively unchanging. From these we computed the average sub-band power within each change-point interval and fitted a Linear Mixed-Effects (LME) regression model to each sub-band with the average sub-band power in each interval as its dependent variable, a fixed effect of time interval as its predictor, and random intercepts for individual sessions. To determine if predicted average sub-band powers vary significantly over time, each sub-band model was compared using a Chi-squared likelihood-ratio test to a corresponding null model that omits its predictor variable. Lastly, to determine how significantly sub-band powers change between detected intervals in each model, we compared average sub-band powers between change-point intervals using a Bonferroni-corrected post hoc pairwise Tukey test.

## 3. Results

**Figure 1:**
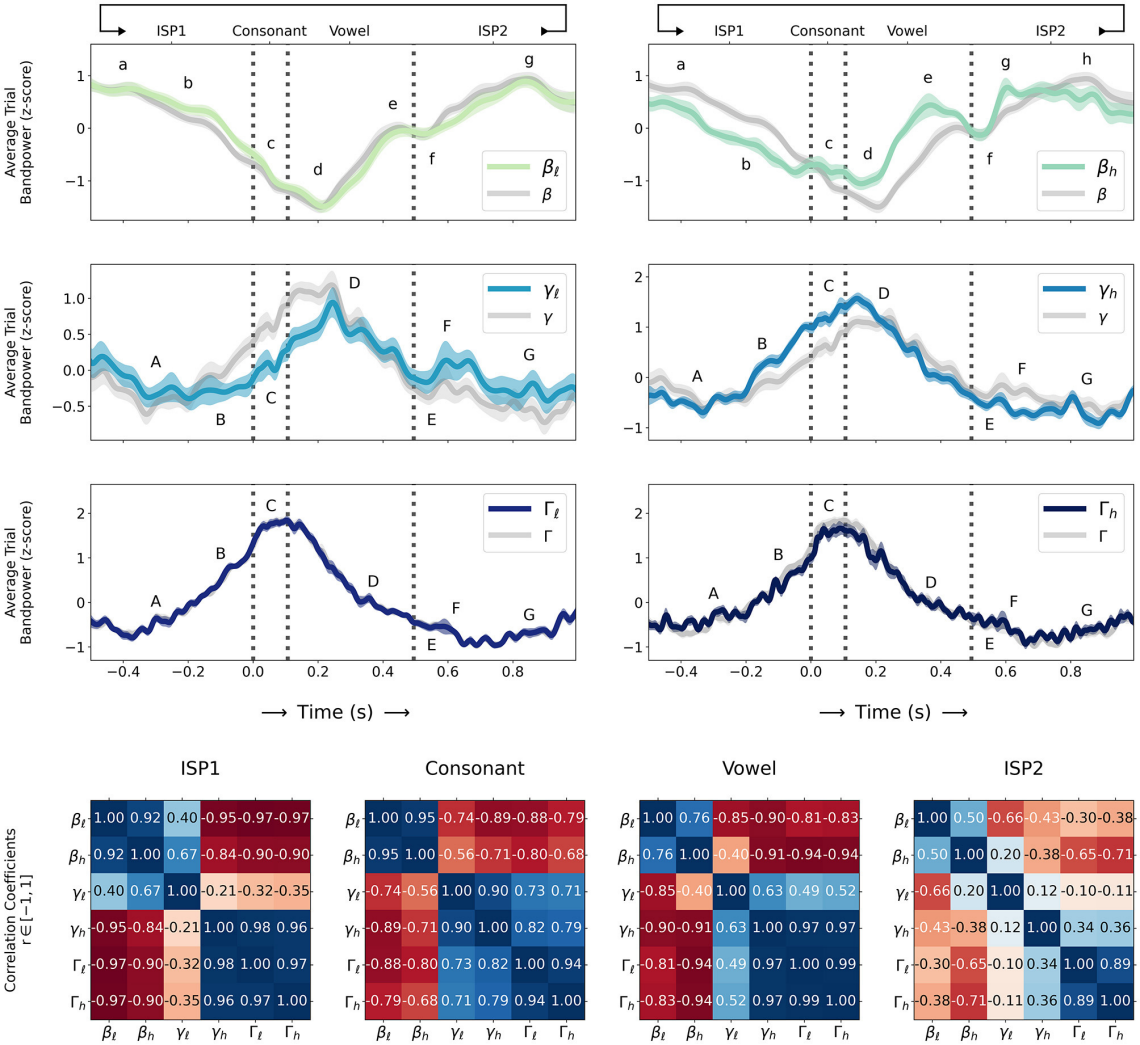
**(Top)** Z-scored trial sub-band powers for β: {β_ℓ_, β_*h*_}, γ: {γ_ℓ_, γ_*h*_}, and Γ: {Γ_ℓ_, Γ_*h*_} (as computed in Section 2.3) during sequences of alternating consonant-vowel speech movement and inter-utterance speech posture (ISP1-CV-ISP2) averaged over all 31 sessions (solid coloured lines; sequential green-blue colour scale for β_ℓ_ through Γ_*h*_ sub-bands) and compared to β, γ, and Γ bandpowers in their entirety (solid grey lines) with confidence intervals (transparent coloured line width) for standard error $\pm \sigma /\sqrt{31}$. Letters **a**-**h** and **A**-**G** label qualitative β and {γ, Γ} sub-band events respectively (as described in Section 3) and consonant movement onset, consonant-vowel transition, and vowel movement termination times are indicated (dashed vertical grey lines); **(Bottom)** Normalized correlation coefficients (numbers in 6x6 grids) computed as Pearson r-values in [−1, 1] (coloured grid squares; diverging red-white-blue colour scale for negative-nil-positive correlations) for pairs of sub-band powers during ISP1, consonant movement, vowel movement, and ISP2 time intervals.

Figure 1 **(top)** shows z-scored trial sub-band powers for β: {β_ℓ_, β_*h*_}, γ: {γ_ℓ_, γ_*h*_}, and Γ: {Γ_ℓ_, Γ_*h*_} (as computed in Section 2.3) during sequences of alternating consonant-vowel speech movement and inter-utterance speech posture (ISP1-CV-ISP2) averaged over all 31 sessions (four subjects) and compared to z-scored average trial β, γ, and Γ bandpowers in their entirety with confidence intervals for standard error $\pm \sigma /\sqrt{31}$, and we label qualitative β and {γ, Γ} sub-band events as follows:

- {β_ℓ_, β_*h*_} (BBA):
  (**a**): starts to decrease from trial maximum before consonant movement onset (during ISP1); BBA decreases in β_*h*_ before β_ℓ_
  (**b**): continues to decrease before consonant movement onset; BBA decreases more rapidly in β_*h*_
  (**c**): briefly increases during consonant movement in β_*h*_; BBA decreases in β_ℓ_
  (**d**): decreases to trial minimum during vowel movement shortly after vowel onset
  (**e**): increases and rapidly peaks before vowel movement termination; BBA peaks differently between β_ℓ_ and β_*h*_
  (**f**): briefly decreases at vowel movement termination
  (**g**): increases again to trial maximum after vowel movement termination (during ISP2); BBA increases more rapidly in β_*h*_
  (**h**): remains at or near trial maximum during ISP2 (β_*h*_ only).
- {γ_ℓ_, γ_*h*_, Γ_ℓ_, Γ_*h*_} (GBA):
  (**A**): starts to increase from trial minimum before consonant movement onset (during ISP1); γ_ℓ_ briefly decreases; GBA increases in γ_*h*_, Γ_ℓ_, Γ_*h*_ before γ_ℓ_
  (**B**): continues to increase before consonant movement onset; GBA increases more rapidly in γ_*h*_, Γ_ℓ_, Γ_*h*_ than γ_ℓ_
  (**C**): peaks to trial maximum in γ_*h*_, Γ_ℓ_, Γ_*h*_; GBA continues to increase in γ_ℓ_ with some oscillation
  (**D**): peaks to trial maximum in γ_ℓ_ shortly after vowel onset; GBA decreases in γ_*h*_, Γ_ℓ_, Γ_*h*_
  (**E**): continues to decrease until after vowel movement termination (during ISP2)
  (**F**): briefly increases in γ_ℓ_ and γ_*h*_; Γ_ℓ_ and Γ_*h*_ decrease to trial minimum
  (**G**): remains at or near trial minimum during ISP2.

**Figure 2:**
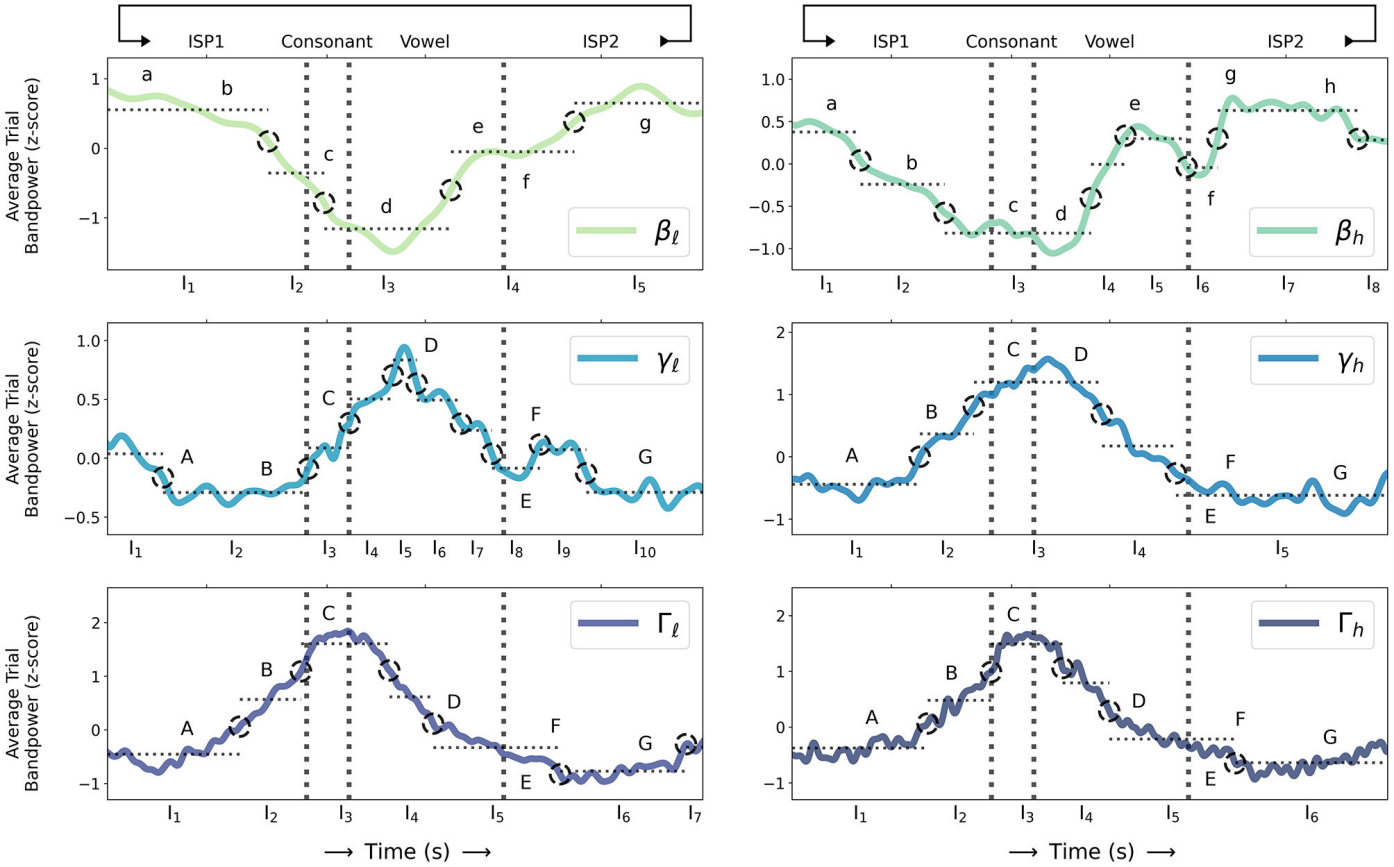
Z-scored trial sub-band powers for β: {β_ℓ_, β_*h*_}, γ: {γ_ℓ_, γ_*h*_}, and Γ: {Γ_ℓ_, Γ_*h*_} (as computed in Section 2.3) during sequences of alternating consonant-vowel speech movement and inter-utterance speech posture (ISP1-CV-ISP2) averaged over all 31 sessions (solid coloured lines; sequential green-blue colour scale for β_ℓ_ through Γ_*h*_ sub-bands) and compared to change points detected from sub-band power statistics (dashed black circles) and average sub-band power during time intervals between pairs of change points (dotted horizontal grey lines) where sub-band power statistics are relatively unchanging. Consonant movement onset, consonant-vowel transition, and vowel movement termination times are indicated (dashed vertical grey lines). Letters **a**-**h** and **A**-**G** label qualitative β and {γ, Γ} sub-band events respectively and are compared to detected change points (as described in Section 3).

Supplementary Table S3 summarizes the LME models fit to each sub-band. A likelihood-ratio test between each sub-band LME model and its corresponding null model shows that sub-band activity varies significantly over time; average BBA and GBA during CV speech movement and ISP intervals are significantly non-constant (*p* < 0.00001 for all sub-bands, see Table 1 for details). Detected change-point intervals and the average sub-band power in each change-point interval are shown in Figure 2, and a post-hoc pairwise Tukey test with a Bonferroni correction between change-point intervals in each model identifies significant changes in average BBA and GBA over the time course of individual speech movements.

**Table 1:** Likelihood-ratio test results for each sub-band LME model and its corresponding null model using a Chi-squared test.

**Sub-band**	**Likelihood-ratio test results**
β_ℓ_	χ^2^ (4) = 187.46, *p* < 2.2*e*−16
β_*h*_	χ^2^ (7) = 90.062, *p* < 2.2*e*−16
γ_ℓ_	χ^2^ (9) = 69.477, *p* < 1.93*e*−11
γ_*h*_	χ^2^ (4) = 198.58, *p* < 2.2*e*−16
Γ_ℓ_	χ^2^ (6) = 305.93, *p* < 2.2*e*−16
Γ_*h*_	χ^2^ (5) = 207.3, *p* < 2.2*e*−16

Degrees of freedom (in parenthesis), Chi-squared values, and p-values are reported.

Both β_ℓ_ and β_*h*_ sub-band powers start to decrease up to 400 ms before consonant movement onset (**a**, **b** as described above; equivalently the end of ISP1; *p* < 2.2*e*−16: = ϵ for β_ℓ_ comparing {I_1_, I_2_} as labelled in Figure 2; *p* < 0.005 for β_*h*_ comparing {I_1_, I_2_}), continue to decrease through consonant movement (**c**; *p* < 0.00001 for β_ℓ_ comparing {I_2_, I_3_}; *p* < 0.01 for β_*h*_ comparing {I_2_, I_3_}), and to their respective trial minima (over all of ISP1, CV movement, and ISP2) during vowel movement (**d**; *p* < 0.00001 for β_ℓ_ comparing I_3_ with all intervals; *p* < 0.01 for β_*h*_ comparing I_3_ with all intervals). BBA increases shortly after vowel onset to a local maximum shortly before vowel movement termination (**d**, **e**; equivalently the start of ISP2; *p* < ϵ for β_ℓ_ comparing {I_3_, I_4_}; *p* < 0.00005 for β_*h*_ comparing {I_3_, I_4_}), and decreases shortly after vowel termination though not significantly in β_*h*_ (**e**, **f**; *p* > 0.9 for β_*h*_ comparing {I_5_, I_6_}), followed by a further increase across {β_ℓ_, β_*h*_} to their respective trial maxima (**f**, **g**; *p* < 0.00001 for β_ℓ_ comparing {I_4_, I_5_}; *p* < 0.001 for β_*h*_ comparing {I_6_, I_7_}), which are sustained in β until subsequent movement (**g**, **h**; *p* > 0.99 for β_ℓ_ comparing {I_1_, I_5_}; *p* > 0.99 for β_*h*_ comparing {I_1_, I_7_}, *p* > 0.99 comparing {I_1_, I_8_}, and *p* > 0.75 comparing {I_7_, I_8_}). Notably there are no significant differences between average β in the first and last change-point intervals in ISP1 and ISP2 respectively.

All of {γ_*h*_, Γ_ℓ_, Γ_*h*_} start to increase up to 300 ms before consonant onset (**A**, **B** as described above; equivalently the end of ISP1; *p* < 0.00001 for γ_*h*_ comparing {I_1_, I_2_}; *p* < ϵ for Γ_ℓ_ comparing {I_1_, I_2_}; *p* < 0.00001 for Γ_*h*_ comparing {I_1_, I_2_}), continue to increase (**B**, **C**; *p* < 0.00001 for γ_*h*_ comparing {I_2_, I_3_}; *p* < ϵ for Γ_ℓ_ comparing {I_2_, I_3_}; *p* < 0.00001 for Γ_*h*_ comparing {I_2_, I_3_}) to their respective trial maxima (**C**; *p* < 0.00001 for γ_*h*_ comparing I_3_ with all intervals; *p* < ϵ for Γ_ℓ_ comparing I_3_ with all intervals; *p* < 0.00001 for Γ_*h*_ comparing I_3_ with all intervals) at vowel onset, and decrease through vowel termination (**D**-**F**; equivalently the start of ISP2; *p* < 0.00001 for γ_*h*_ comparing {I_4_, I_5_}; *p* < ϵ for Γ_ℓ_ comparing {I_4_, I_5_}; *p* < 0.00001 for Γ_*h*_ comparing {I_4_, I_5_}). Like β there are no significant differences between average {γ_*h*_, Γ_ℓ_, Γ_*h*_} in the first and last change-point intervals in ISP1 and ISP2 respectively (*p* > 0.75 for γ_*h*_ comparing {I_1_, I_5_}; *p* > 0.05 for Γ_ℓ_ comparing {I_1_, I_6_}; *p* > 0.5 for Γ_*h*_ comparing {I_1_, I_6_}).

Conversely γ_ℓ_ briefly decreases though not significantly before consonant movement onset (**A**, **B**; *p* > 0.99 comparing {I_1_, I_2_, I_3_}), does not significantly increase until consonant onset (**C**, **D**; *p* > 0.99 comparing {I_2_, I_3_}; *p* > 0.75 comparing {I_3_, I_4_}; *p* < 0.0005 comparing {I_2_, I_4_}), increases to its trial maximum during *mid*-vowel movement (**D**; *p* < 0.00001 comparing {I_2_, I_5_}; *p* < 0.00001 comparing {I_5_, I_8_}; *p* > 0.99 comparing {I_2_, I_8_}), decreases through vowel movement termination (**D**-**F**; *p* < 0.05 comparing {I_5_, I_7_}; *p* < 0.00001 comparing {I_5_, I_8_}), and increases again shortly though not significantly after vowel termination (**F**; *p* > 0.99 comparing {I_8_, I_9_, I_10_}). Like {γ_*h*_, Γ_ℓ_, Γ_*h*_} there is no significant difference between average γ_ℓ_ in ISP1 and ISP2 (*p* > 0.99 comparing {I_2_, I_10_}).

**Figure 3:**
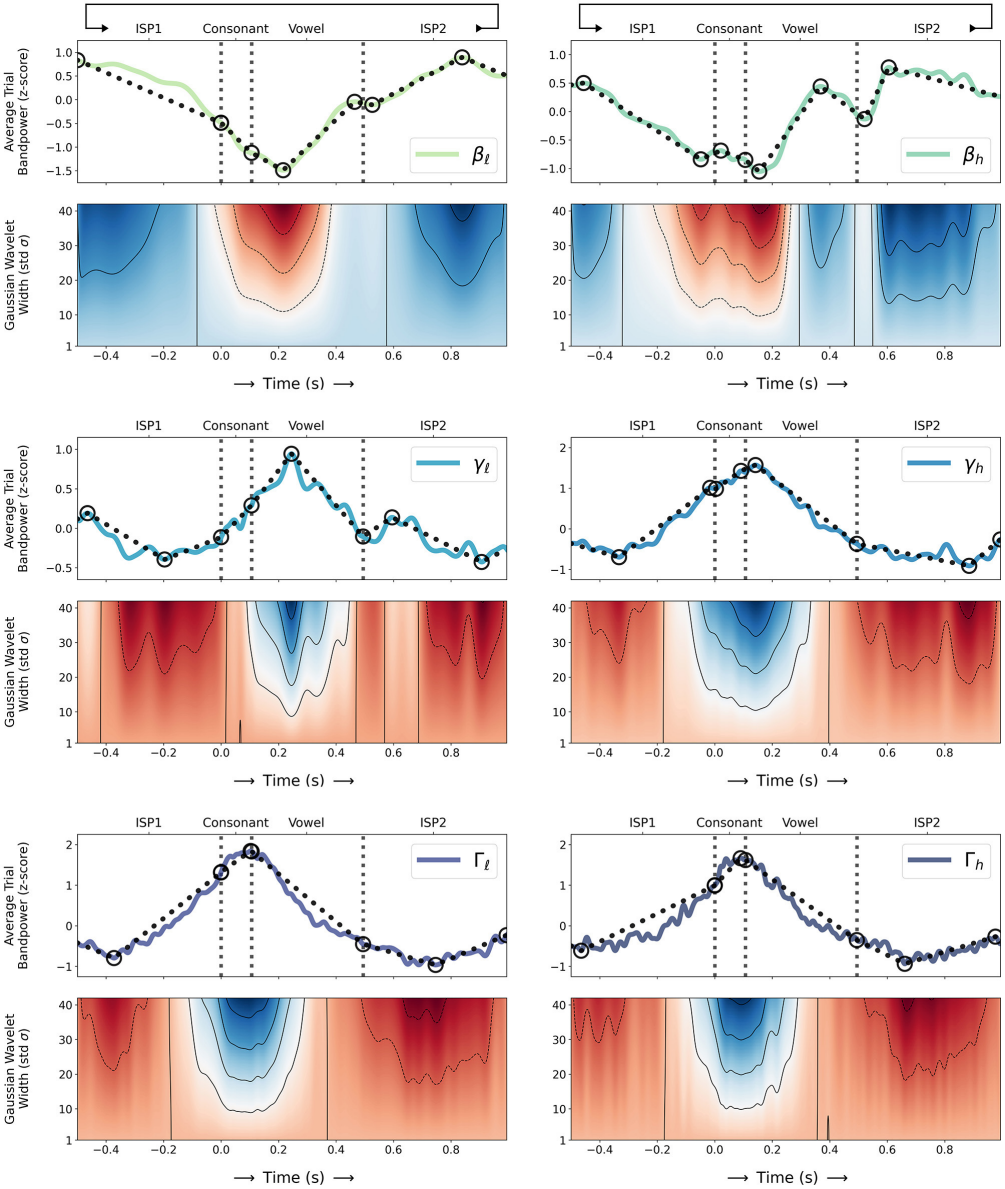
**(Top)** Z-scored trial sub-band powers for β: {β_ℓ_, β_*h*_}, γ: {γ_ℓ_, γ_*h*_}, and Γ: {Γ_ℓ_, Γ_*h*_} (as computed in Section 2.3) during sequences of alternating consonant-vowel speech movement and inter-utterance speech posture (ISP1-CV-ISP2) averaged over all 31 sessions (solid coloured lines; sequential green-blue colour scale for β_ℓ_ through Γ_*h*_ sub-bands) and compared to local sub-band power maxima and minima in ISP1, consonant movement, vowel movement, and ISP2 time intervals (solid and dashed circles respectively). Linearly connected maxima and minima (dotted black lines) show local upward and downward trends and oscillations in BBA and GBA over the time course of individual speech movements; **(Bottom)** Wavelet transforms of average trial sub-band powers using Gaussian wavelets of varying width (standard deviation; σ = 1 to 42) show local maxima and minima as denser contours (solid black lines) and correlations and anti-correlations of Gaussian wavelets—resembling peaks—to sub-band power oscillations (diverging red-white-blue colour scale for negative-nil-positive correlations) over time; **(Both)** Consonant movement onset, consonant-vowel transition, and vowel movement termination times are indicated (dashed vertical grey lines).

Figure 3 shows the same z-scored average trial sub-band powers compared to local sub-band power maxima and minima in ISP1, consonant movement, vowel movement, and ISP2 time intervals. Both β ERD and {γ, Γ} sub-band ERS events are qualitatively evident over the time course of individual speech movements. Linearly connected maxima and minima in each sub-band show local upward and downward trends and oscillations in BBA and GBA, and β and respective {γ, Γ} minima and maxima coincide at consonant movement onset, during mid-vowel movement, and at vowel movement termination; {β_ℓ_, β_*h*_} sub-band powers generally decrease where {γ, Γ} sub-band powers increase. Trendlines also show qualitative evidence of two distinct peaks of γ_ℓ_ ERS—namely sub-band ERPs for individual transient movements as observed by Chartier et al. (2018) and Salari et al. (2018)—as local maxima in γ_ℓ_ during mid-vowel movement and immediately after vowel termination. Oscillations in γ_ℓ_ and {γ_*h*_, Γ_ℓ_, Γ_*h*_} show differences toward consonant onset but both trend upward to peak after consonant onset then downward toward vowel termination; {γ_*h*_, Γ_ℓ_, Γ_*h*_} reach their respective trial maxima during consonant movement whereas γ_ℓ_ reaches its trial maximum during mid-vowel movement.

Wavelet transforms of z-scored average trial sub-band powers in Figure 3 using Gaussian wavelets of varying width (standard deviation; σ = 1–42) show local maxima and minima as denser contours and correlations and anti-correlations of Gaussian wavelets—resembling peaks—to sub-band power oscillations over time. Wavelets confirm local upward and downward trends and oscillations in BBA and GBA; denser contours coincide reasonably well with identified local maxima and minima for each sub-band at consonant onset, during mid-vowel movement, and at vowel termination, and larger Gaussian wavelets correlate or anti-correlate compatibly to β, γ, and Γ oscillations over the time course of speech movements. Wavelets also show two distinct peaks of {γ_ℓ_, γ_*h*_} ERS during (0 < *t* < 500 ms) and immediately after speech movement (600 < *t* < 700 ms) supporting similar observations by Chartier et al. (2018) and Salari et al. (2018), and a local minimum in β_*h*_ immediately after vowel termination (500 < *t* < 550 ms).

Normalized correlation coefficients in Figure 1 **(bottom)** computed as Pearson r-values in [−1, 1] for pairs of sub-band powers during ISP1, consonant movement, vowel movement, and ISP2 time intervals show significant correlations and anti-correlations (0 < *r* < 1 and −1 < *r* < 0 respectively) between {β_ℓ_, β_*h*_} and {γ, Γ} sub-bands (*p* < 0.00005≪α_*critical*_ for all intervals; largest p-values between γ_ℓ_ and {γ_*h*_, Γ_ℓ_, Γ_*h*_} during ISP2; smallest p-values between all sub-bands during vowel movement). Particularly there are strong anti-correlations (−1 < *r*≪0) between β and Γ sub-band powers during both consonant (−0.88 < *r* < −0.68; *p* < ϵ) and vowel movement (−0.94 < *r* < −0.81; *p* < ϵ), reasonably strong anti-correlations between β and γ sub-band powers (consonant: −0.89 < *r* < −0.56; vowel: −0.91 < *r* < −0.4; *p* < ϵ for both), strong anti-correlations between β and {γ_*h*_, Γ_ℓ_, Γ_*h*_} during ISP1 (−0.97 < *r* < −0.84; *p* < ϵ) and weaker anti-correlations between β and {γ_*h*_, Γ_ℓ_, Γ_*h*_} during ISP2 (−0.71 < *r* < −0.3; *p* < ϵ) with the exception of a weak but positive correlation between β_*h*_ and γ_ℓ_ (*r* = 0.2; *p* < 0.00001). There are also positive correlations between {β_ℓ_, β_*h*_} and γ_ℓ_ in ISP1 (0.4 < *r* < 0.67; *p* < ϵ) and stronger positive correlations between {γ, Γ} sub-bands during consonant (0 ≪ 0.71 < *r* < 1; *p* < ϵ) and vowel movement (0 < 0.49 < *r* < 1; *p* < ϵ).

Notably such anti-correlations in a fine motor context (as speech) are consistent with beta desynchronization findings in non-speech gross motor contexts by Engel and Fries (2010) and Kilavik et al. (2013), and there are different anti-correlations between β and {γ, Γ} sub-bands over the time course of speech movements: strong anti-correlations between β and Γ during ISP1 (*r* < −0.9; before movement onset) weaken during consonant movement (−0.88 < *r* < −0.68), tend to stronger anti-correlations between β and γ (−0.89 < *r* < −0.56), strengthen again between β and Γ during vowel movement (*r* < −0.81), and finally weaken but remain anti-correlated with β across {γ_*h*_, Γ_ℓ_, Γ_*h*_} during ISP2 (−0.71 < *r* < −0.3; after movement termination).

## 4. Discussion

We hypothesized that sensorimotor β oscillations during speech and ISP reflect motor inhibition and set maintenance, and compared β, γ, and Γ activity in the SMC during speech and inter-utterance rest using high-density ECoG. We showed that inhibitory BBA modulates the termination of individual phonemic movements throughout the articulation of a syllable, and contributes to maintenance of tonic postural states during ISP. Specifically our hypothesis bore five predictions, and all were supported by our results as follows:

1. The onset of movement resulted in a measurable decrease in BBA (ERD).
2. The termination of movements resulted in a measurable increase in BBA (ERS).
3. Maintenance of vocal tract posture corresponded with sustained BBA.
4. γ and Γ corresponded with transient movement into the target rest posture (ISP).
5. β and {γ, Γ} activity were anti-correlated during movement execution and postural maintenance.

For predictions 1 and 2, the articulation of a single syllable is the product of multiple discrete movements, and was thus predicted to involve multiple instances of movement onset and termination. As predicted, multiple cycles of ERD and ERS were observed across {β_ℓ_, β_*h*_} corresponding with each discrete movement. As our results supported all five predictions, we note three major observations, each presented in detail within its relevant subsection.

### 4.1. Speech movement onset, maintenance, and termination are reflected in BBA

We observed that BBA is responsive to the onset and termination of individual movements within a sequence. Notably, a reduction to BBA commenced 400 ms before speech movement onset (“**a**” as described in Section 3) and continued decreasing until the CV transition (**a**-**d**), where BBA increased in the higher-frequency sub-band (β_*h*_; **c**). BBA subsequently decreased during the initial execution of the vowel (**d**), but rose again shortly before the vowel midpoint until shortly before movement termination (**d, e**). The rise in BBA mid-vowel was unexpected, as we expected that BBA would remain suppressed during the execution of movement. However, we consider that while speech sounds such as consonants are composed of rapid transient movements, vowels involve sustained postural configurations that are maintained along longer time-courses (Salari et al., 2018). Thus, BBA appears to underlie the maintenance of postural speech movement targets. Further supporting this interpretation is the observation that {γ, Γ} decreased before the vowel midpoint until movement termination (**D, E**) in correspondence with increase of β. BBA continued to rise until vowel termination, where it then decreased when ISP return movement was expected (**f**), and then subsequently rose during maintenance of ISP (**g, h**). Additional experiments comparing BBA across vowels sustained for varying lengths would be beneficial for confirming the role of β in maintaining postural speech movements.

### 4.2. Low and high-frequency β correspond to similar motor functions

We decomposed β into low and high sub-bands to investigate whether they correspond to distinct motor functions, namely inhibition and maintenance versus planning. We note that qualitatively both sub-bands {β_ℓ_, β_*h*_} followed the same general trends, and thus do not appear to support distinct motor functions within sub-bands. Both sub-bands appear responsive to individual movements within the CV sequence; for example, both β_ℓ_ and β_*h*_ appear to maintain vowel phonation (**d, e**). Yet only β_*h*_ appears to directly inhibit individual phonemic movements within the sequence, as observed by an increase in β_*h*_ during consonant execution where inhibition is necessary (**c**), and a decrease where release of inhibition is necessary for movement during ISP return (**f**). This may be due to the longer time course of one cycle of β_ℓ_ (~48–84 ms) obscuring changes over a short movement time course (~107 ms consonant). Thus, there is no strong evidence that β_ℓ_ and β_*h*_ modulate different functions of inhibition and maintenance versus movement planning; however, it is possible that β_*h*_ corresponds to inhibition in a manner not observed in β_ℓ_. We would expect if β_*h*_ corresponds exclusively to a system of movement planning, such as that described by De Nil et al. (2021), that individual movements within a sequence would not result in notable β oscillations; β oscillations were reported only at the beginning and end of sequences of syllables (De Nil et al., 2021). However, the fact that β_*h*_ corresponded directly with individual phonemic units strongly suggests that a system of state maintenance and inhibition must be considered alongside any role of β in the planning of motor sequences.

Differences in recording mediums may also contribute to discrepancies between the present work and those of De Nil et al. (2021); our study used ECoG recorded from the SMC, which benefits from localized placement and thus high spatiotemporal resolution (Siero et al., 2014), whereas De Nil et al. used MEG, which does not have the same localization as ECoG. Direct comparisons of MEG and ECoG for detection of BBA and GBA reveal that while MEG is generally consistent with ECoG, many small-scale changes in neural activity are observable only with ECoG (Dalal et al., 2008). For example, approximately half of all spikes observed in ECoG have a detectable MEG counterpart (Huiskamp et al., 2010).

Overall, these findings demonstrate that BBA is modulated not only by holistic speech actions, such as the articulation of syllables, but also by individual speech movements.

We note that observations by Livezey et al. (2019) of positive correlations (coupling) between β and Γ during CV transitions are inconsistent with our findings, as Γ and β were largely anti-correlated during consonant movement, which suggests that any coupling of β and Γ during CV transitions should reflect independent but rather negatively-coupled Γ-activation and β-inhibition ERPs executed at the pace of rapid sequential speech units. Indeed, the positive coupling that Livezey et al. (2019) report is band-limited to β frequencies near 23 Hz (within β_*h*_) and other β frequencies show nil or negative correlations instead (as described in Section 3). This said, we were able to synthetically reproduce the positive Γ–β correlation that they report by computing Γ–β correlations (0.43 < *r* < 0.81; *p* < ϵ⋘α_*critical*_) over a period of consonant movement centered at CV transition to reflect this part of their methodology (for a comparison of Pearson r-values, see Supplementary Figure S2). This confirms Livezey et al. (2019)'s observation that coupling effects are limited to specific sub-bands of oscillations, and supports their observation that analysis of specific sub-bands produces a more nuanced representation of neural oscillations. However, when comparing BBA and GBA along the rapid time course of individual CV speech movements in sequence—as opposed to focusing on the CV transition as Livezey et al. do—we see only negative Γ–β coupling resembling previous beta desynchronization findings in gross motor contexts (Engel and Fries, 2010; Kilavik et al., 2013).

### 4.3. γ/Γ reflect movement, β reflects maintenance and inhibition

Overall, γ and Γ oscillations were similar throughout movement execution and ISP, although individual ERPs were most salient in γ. We observed distinct γ ERPs corresponding to both the execution of phonemic targets (**C**, **D**) and the return movement to ISP (**F**). This provides neurological evidence for similarities between movement control into both phonemic targets and ISP as described by Gick et al. (2004). Notably, instances of lower-frequency GBA were consistent in time and number with major beta desynchronization events, which was reflected in a general trend of anti-correlation between γ/Γ and β. A small transient spike across {γ_ℓ_, γ_*h*_} was also observed after vowel termination corresponding with Salari et al. (2018)'s own observations of GBA during movement into ISP. Subsequently {γ, Γ} continued to decrease throughout maintenance of ISP to their lowest values (**F**, **G**). Conversely, β spiked at the start of ISP and remained elevated until the onset of subsequent movement (**g, a**). This is consistent with previous descriptions of ISP movement and control, as well as theories of BBA as a general mechanism for maintaining status quo (Engel and Fries, 2010; Kilavik et al., 2013).

### 4.4. Limitations

We only investigated ECoG signals corresponding to CV syllables containing /i/, so we did not investigate the influence of task complexity on β oscillations and fine motor postural control. Further studies could examine BBA patterns of ISP with repeating sequences of simple syllables (e.g., /pa pa pa/) versus non-repeating sequences of complex syllables (e.g., /pa ta ka/). Furthermore, we note that De Nil et al. (2021) compared β oscillations over longer rest periods allowing insight into BBA distinct from potentially interfering motor activity such as {γ, Γ} spiking and/or ERS. While the present work demonstrates BBA as being modulated by motor activity, further analysis over longer rest periods is necessary to understand how BBA may respond to components of motor planning such as sequence complexity.

We expected based on descriptions by Salari et al. (2018) that Γ would be maintained throughout vowel execution. Instead, we saw higher-frequency GBA decrease throughout vowel execution while BBA increased. This suggests to us that β modulation is necessary for maintenance of tonic states in speech movements. However, this needs to be verified by analysis of BBA during the articulation of sustained vowels of various lengths.

The influence of postural complexity on BBA was also not investigated; further studies could explore β oscillations in fine motor postural maintenance within setups that require varying postural complexity, for example using orientations agonistic or antagonistic to gravity (upright, supine, or upside-down via an inversion table). Biomechanical simulations suggest that increased vocal tract postural complexity necessitates increased tonic muscle activation (Liu et al., 2022), which suggests in turn that increased BBA will be observed in correspondence with increased postural complexity.

Finally, we assume that speakers return to ISP between utterances based on previous observations of universal human motor behavior in speech contexts. The interstimulus interval (~ 820 ms; the median length of included inter-speech intervals) in our experiment is consistent with experiments where ISP is observed and reported (Gick et al., 2004; Ramanarayanan et al., 2014; Gick and Mayer, 2019). However, Bouchard and Chang (2019) do not provide a direct measurement of articulator position throughout speech and inter-speech periods. While we have no reason to expect unusual motor behavior during rest, ideally future experiments will directly correlate articulator positions to BBA and GBA.

## 5. Conclusion

We used electrocorticography to assess the contribution of sensorimotor beta-band activity during speech articulation and postural maintenance to understand the neural control of postural states in the vocal tract. We saw strong anti-correlations of β and {γ, Γ} oscillations during speech movements and demonstrated correspondence of β to inhibition of discrete speech movements and maintenance of tonic postural states in the vocal tract. We decomposed β into low and high-frequency sub-bands to investigate whether they correspond to distinct motor functions and found no strong evidence of functional differentiation between β sub-bands. However, β_*h*_ showed more evidence of inhibition of individual phonemic movements. These findings identify consistencies between the neural control of posture in speech—a fine motor skill—and what has been previously reported for gross motor skills (Schmidt et al., 2019). The consistency of β in relation to fine motor skills and gross motor skills provides support for a unified model of postural control across gross and fine motor domains.

## Data availability statement

The datasets presented in this study can be found in online repositories. The names of the repository/repositories and accession number(s) can be found at: https://www.github.com/ericeasthope/as-in-ecog and https://figshare.com/collections/Human_ECoG_speaking_consonant-vowel_syllables/4617263.

## Author contributions

EE contributed electrocorticography (ECoG) data pre-processing, signal analysis, project code, data visualizations, and wrote the methods and results. AS contributed project design, literature review, theoretical background, data interpretation, and wrote the introduction and discussion. YL assisted with project planning and contributed statistical analysis. BG and SF provided theoretical and methodological supervision and are the funding authors. All authors contributed to the article and approved the submitted version.
