# Supplementary material for: Cortical control of posture in fine motor skills: evidence from inter-utterance rest position
Source: Front Hum Neurosci. 2023 Aug 17;17:1139569. doi: 10.3389/fnhum.2023.1139569 (PMC10469778; doi:10.3389/fnhum.2023.1139569)
Supplement: Supplementary file 1 [file Data_Sheet_1.pdf]

SUPPLEMENTARY MATERIAL

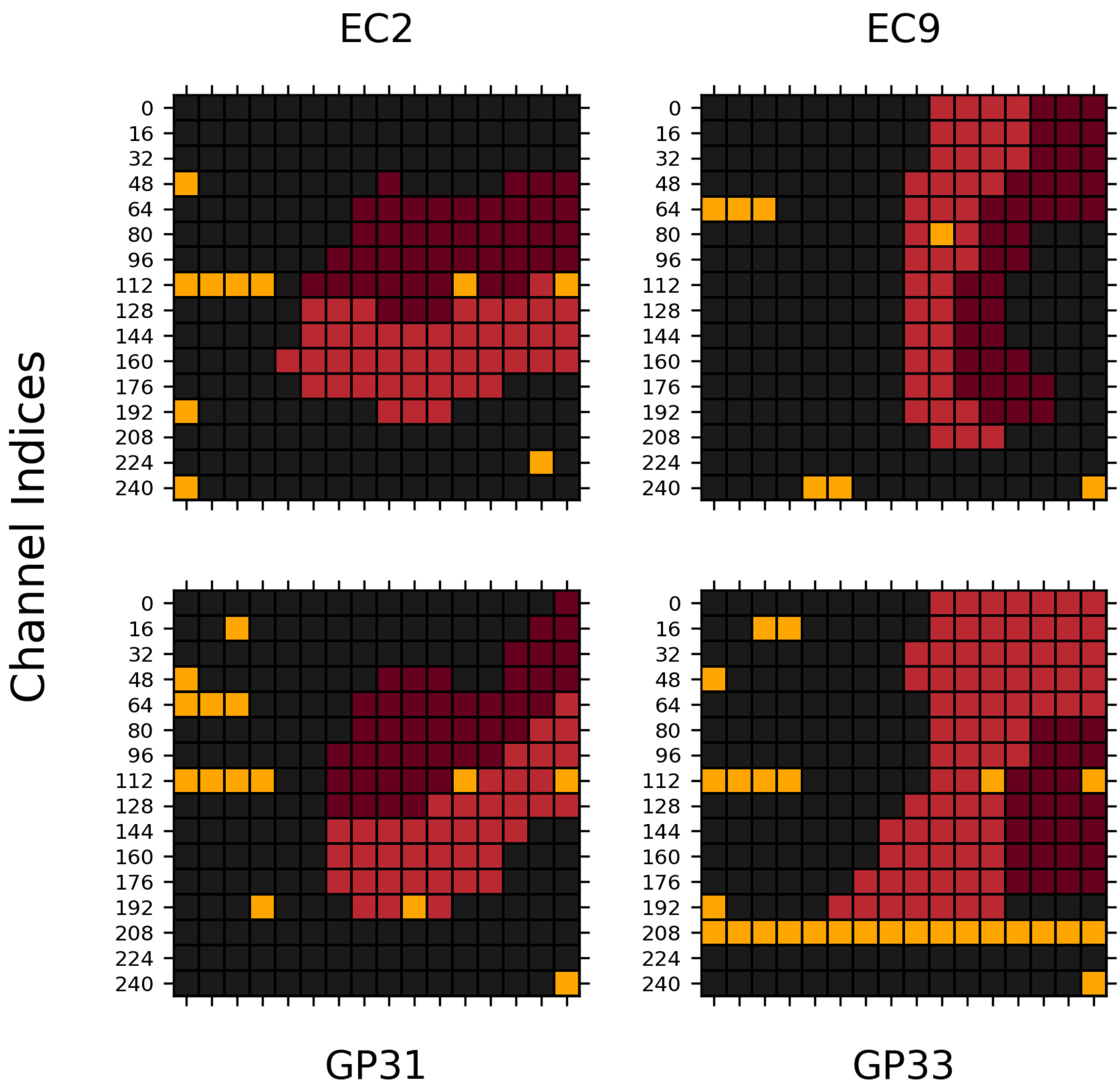

Figure S1. Subject channel indices visualized as coloured squares on four 256-channel grids labelled by subject ID. Valid (non-noisy and non-*ictal*) SMC channels are coloured shades of red, channels outside the SMC are coloured grey, and channels are coloured orange if they are marked invalid ("bad") by Bouchard and Chang (2019) in one or more subject sessions (for SMC channel indices, see Table S1 ; for "bad" channel indices, see Table S2). Channel indices appear spatialized as pre-central (light red) and post-central gyrus (dark red) indices are consistently adjacent and share a common edge (presumably the central sulcus).

Table S1. Subject IDs, SMC channel indices corresponding to ECoG electrodes implanted over the pre-central and post-central gyrus (from locations labelled by Bouchard and Chang (2019)), and the total number of SMC channels (out of 256) per subject.

Subject	**SMC indices**: `[location == "precentral" AND location == "postcentral"]`	Total
EC2	56, 61-63, 71-79, 87-95, 102-111, 117-127, 133-143, 149-159, 164-175, 181-188, 200-202	86
EC9	9-15, 25-31, 41-47, 56-63, 72-79, 88-92, 104-108, 120-123, 136-139, 152-155, 168-172, 184-189, 200-205, 217-219	111
GP31	15, 30-31, 45-47, 56-58, 61-63, 71-79, 87-95, 102-111, 118-127, 134-143, 150-157, 166-172, 182-188, 199-202	107
GP33	9-15, 25-31, 40-47, 56-63, 73-79, 89-95, 105-111, 121-127, 136-143, 151-159, 167-175, 182-191, 197-203, 213-214	111

Table S2. Subject IDs, corresponding session IDs, and invalid ("bad") channel indices marked by Bouchard and Chang (2019) as having noise and/or seizure-related (*ictal*) artifacts during session recordings. Bad channels are excluded from SMC channels by session.

Subject	Session	**Bad indices**: `[bad == True]`
EC2	B1	48, 112-115, 123, 127, 255
EC2	B105	48, 112-115, 123, 127, 255
EC2	B15	48, 112-115, 123, 127
EC2	B76	48, 112-115, 123, 127, 192, 238, 240
EC2	B8	48, 112-115, 123, 127
EC2	B89	48, 112-115, 123, 127, 192, 238, 240
EC2	B9	48, 112-115, 123, 127, 192, 240
EC9	B15	48, 112-115, 123, 127, 192, 240
EC9	B39	89
EC9	B46	89
EC9	B49	89, 244-245, 255
EC9	B53	64-66, 89, 255
EC9	B60	89, 255
EC9	B63	64-66
GP31	B1	18, 48, 112-115, 123, 127, 255
GP31	B2	18, 48, 64-66, 112-115, 123, 127, 255
GP31	B21	48, 64-66, 112-115, 123, 127, 255
GP31	B4	18, 48, 112-115, 123, 127, 255
GP31	B6	18, 48, 112-115, 123, 127, 255
GP31	B63	48, 112-115, 123, 127, 255
GP31	B65	48, 112-115, 123, 127, 255
GP31	B67	48, 112-115, 123, 127, 255
GP31	B69	48, 112-115, 123, 127, 255
GP31	B71	48, 112-115, 123, 127, 255
GP31	B78	48, 64, 66, 112-115, 123, 127, 255
GP31	B82	48, 64, 66, 112-115, 123, 127, 195, 201, 255
GP31	B83	48, 64, 66, 112-115, 123, 255
GP31	B9	48, 64-66, 112-115, 123, 127, 255
GP33	B1	48, 112-115, 123, 127, 192, 255
GP33	B30	18-19, 48, 112-115, 123, 127, 208-223, 255
GP33	B5	48, 112-115, 123, 127, 255

Table S3. LME model summary for all sub-bands. For each sub-band, the average sub-band power in the first interval is used as a reference to estimate relative average sub-band power in all other intervals.

**Sub-band**	**Interval**	**Estimate**	**Std. error**	**Wald statistic** (pseudo t-statistic)
β_ℓ	1	0.55546	0.07727	7.188
β_ℓ	2	-0.91112	0.10928	-8.338
β_ℓ	3	-1.71401	0.10928	-15.685
β_ℓ	4	-0.60528	0.10928	-5.539
β_ℓ	5	0.09441	0.10928	0.864
β_h	1	0.37777	0.11376	3.321
β_h	2	-0.61862	0.16089	-3.845
β_h	3	-1.19318	0.16089	-7.416
β_h	4	-0.38154	0.16089	-2.371
β_h	5	-0.0791	0.16089	-0.492
β_h	6	-0.42149	0.16089	-2.62
β_h	7	0.25325	0.16089	1.574
β_h	8	-0.09488	0.16089	-0.59
γ_ℓ	1	0.03785	0.12333	0.307
γ_ℓ	2	-0.3273	0.17442	-1.876
γ_ℓ	3	-0.32954	0.17442	-1.889
γ_ℓ	4	0.05042	0.17442	0.289
γ_ℓ	5	0.46552	0.17442	2.669
γ_ℓ	6	0.79663	0.17442	4.567
γ_ℓ	7	0.45688	0.17442	2.619
γ_ℓ	8	0.19941	0.17442	1.143
γ_ℓ	9	-0.12315	0.17442	-0.706
γ_ℓ	10	0.0354	0.17442	0.203
γ_h	1	-0.43869	0.07173	-6.116
γ_h	2	0.80814	0.10144	7.967
γ_h	3	1.63487	0.10144	16.117
γ_h	4	0.61492	0.10144	6.062
γ_h	5	-0.17618	0.10144	-1.737
Γ_ℓ	1	-0.45242	0.07877	-5.744
Γ_ℓ	2	1.02034	0.11139	9.16
Γ_ℓ	3	2.05903	0.11139	18.484
Γ_ℓ	4	1.06775	0.11139	9.585
Γ_ℓ	5	0.1242	0.11139	1.115
Γ_ℓ	6	-0.31542	0.11139	-2.832
Γ_ℓ	7	0.13047	0.11139	1.171
Γ_h	1	-0.37206	0.09279	-4.01
Γ_h	2	0.85237	0.13122	6.496
Γ_h	3	1.86449	0.13122	14.209
Γ_h	4	1.16497	0.13122	8.878
Γ_h	5	0.15919	0.13122	1.213
Γ_h	6	-0.26525	0.13122	-2.021

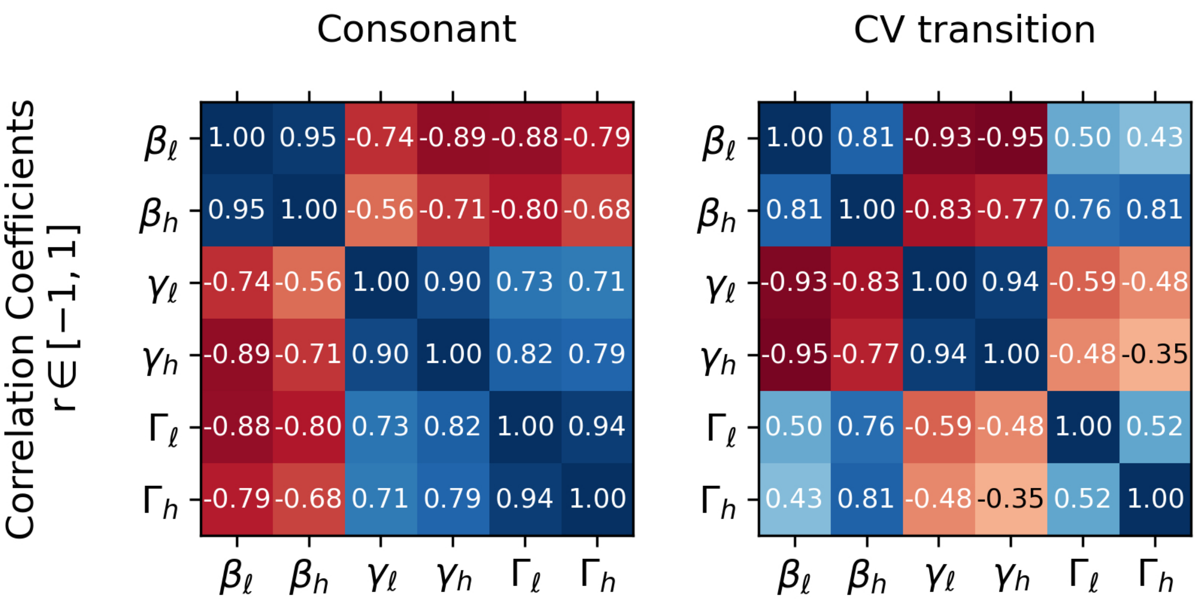

Figure S2. **(Left)** Sub-band correlations in the consonant interval (computed in Easthope et al. (2023), Section 2) as normalized Pearson r-values in $[-1, 1]$. The same correlations are shown in Figure 1; **(Right)** Sub-band correlations in a consonant-length (approx. 107 ms) interval centred at CV transition. Computing correlations centred at CV transition shows positive Γ–β coupling similar to what Livezey et al. (2019) report for β frequencies near 23 Hz, but across both sub-bands $\{\beta_\ell, \beta_h\}$ with stronger correlations between β_h and Γ. Yet when correlations are computed over intervals of rapid individual speech movements this effect seems to vanish (Figure 1), resembling beta desynchronization findings in gross motor contexts (Kilavik et al., 2013; Engel and Fries, 2010).

REFERENCES

[Dataset] Bouchard, K. E. and Chang, E. F. (2019). Human ECoG speaking consonant-vowel syllables. doi:10.6084/m9.figshare.c.4617263.v4

Easthope, E., Shamei, A., Liu, Y., Gick, B., and Fels, S. (2023). Cortical control of posture in fine motor skills: Evidence from inter-utterance rest position. *Frontiers in Human Neuroscience* 17. doi:10.3389/fnhum.2023.1139569

Engel, A. K. and Fries, P. (2010). Beta-band oscillations — signalling the status quo? *Current Opinion in Neurobiology* 20, 156–165. doi:10.1016/j.conb.2010.02.015

Kilavik, B. E., Zaepffel, M., Brovelli, A., MacKay, W. A., and Riehle, A. (2013). The ups and downs of beta oscillations in sensorimotor cortex. *Experimental Neurology* 245, 15–26. doi:10.1016/j.expneurol.2012.09.014

Livezey, J. A., Bouchard, K. E., and Chang, E. F. (2019). Deep learning as a tool for neural data analysis: Speech classification and cross-frequency coupling in human sensorimotor cortex. *PLOS Computational Biology* 15, e1007091. doi:10.1371/journal.pcbi.1007091
